# Supplementary material for: Ancient Fennoscandian genomes reveal origin and spread of Siberian ancestry in Europe
Source: Nat Commun. 2018 Nov 27;9:5018. doi: 10.1038/s41467-018-07483-5 (PMC6258758; doi:10.1038/s41467-018-07483-5)
Supplement: Supplementary file 1 — Supplementary Information [file 41467_2018_7483_MOESM1_ESM.pdf]

Supplementary Material for Ancient Fennoscandian genomes reveal origin and spread of Siberian ancestry in Europe

Supplementary Figures

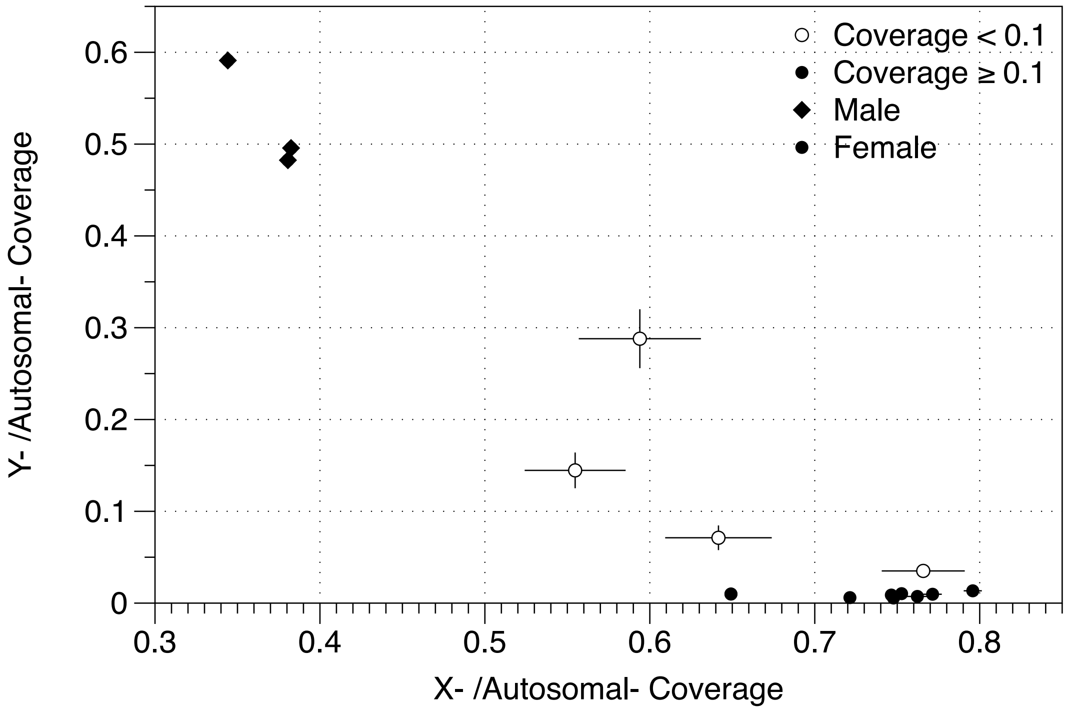

Supplementary Figure 1. Sex Determination. X-chromosomal coverage vs. Y-chromosomal coverage, normalised by autosomal coverage. Low autosomal coverage individuals (n = 4, excluded from downstream analyses) are shown as empty circles, and higher coverage individuals (n = 11) as filled circles. Error bars represent the uncertainty in the calculation of relative coverages, assuming reads map on the capture targets randomly (see Supplementary Text 2 for details). Source data are provided as a Source Data file.

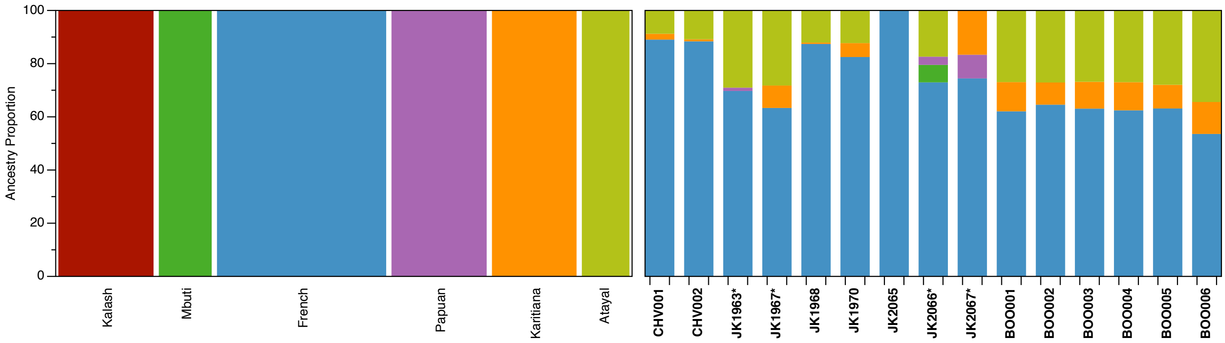

Supplementary Figure 2. Supervised ADMIXTURE. Individuals excluded from further analyses due to low coverage are signified with an asterisk. Among higher coverage genomes, the results within each population are homogeneous, with the exception of the outlier JK2065 within Levänluhta. Source data are provided as a Source Data file.

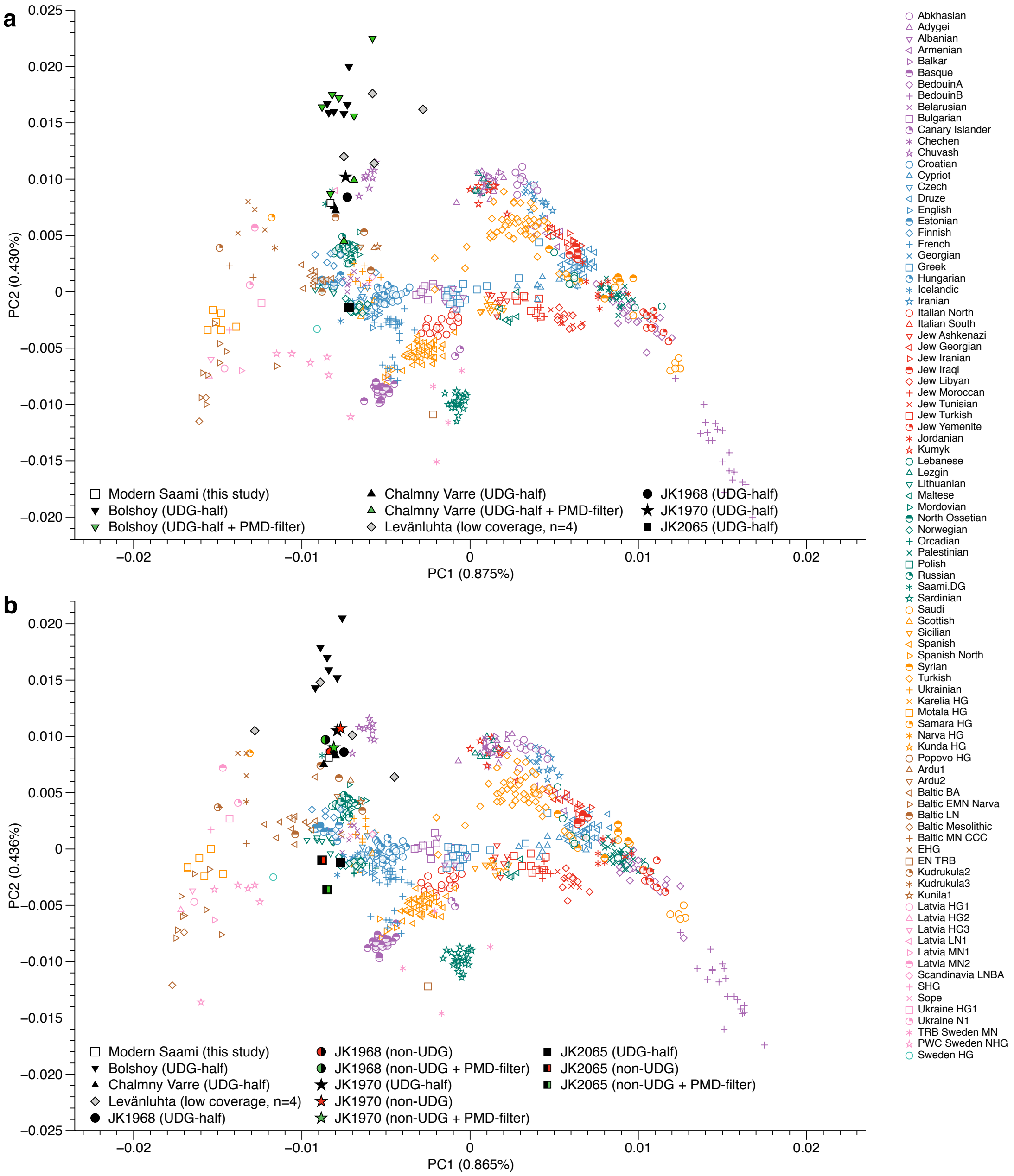

Supplementary Figure 3. Comparison of projection before and after PMD-filtering in PCA space. **a** PCA plot of Europe with individuals from this study projected on principal components constructed on the modern populations in the legend, using the option "shrinkmode: YES". For each individual from Bolshoy and Chalmny Varre, an additional projection is shown for the PMD-filtered dataset. Ancient individuals with fewer than 15,000 covered SNPs are shown in grey. **b** PCA plot of Europe with individuals from this study projected on principal components constructed using only transversion SNPs of the modern populations in the legend, using the option "shrinkmode: YES". For the three Levänluhta individuals with more than 15,000 covered SNPs, additional point for the PMD-filtered and the non-filtered datasets of the non-UDG treated libraries is shown. Ancient individuals with fewer than 15,000 covered SNPs are shown in grey. Source data are provided as a Source Data file.

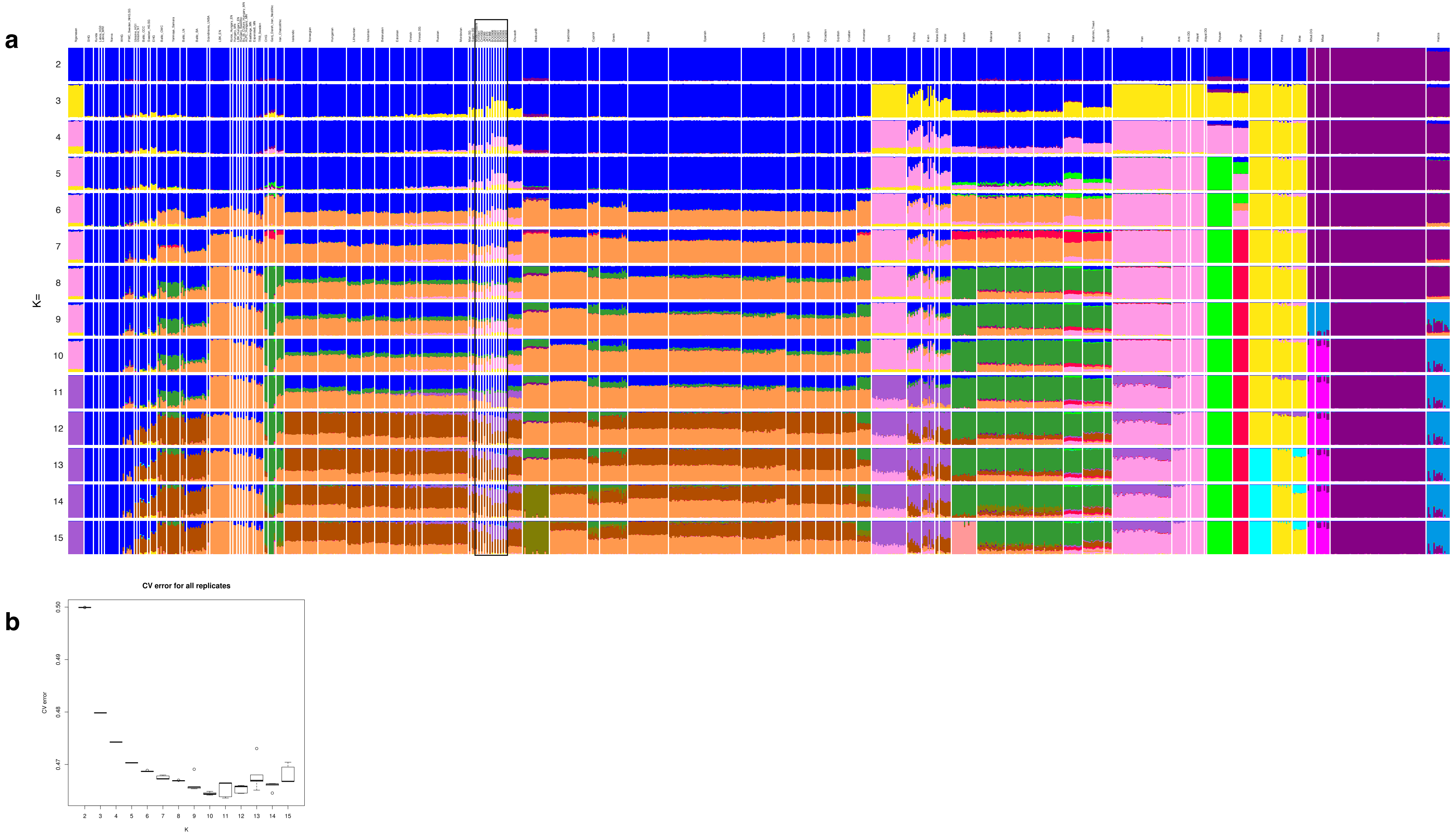

Supplementary Figure 4. ADMIXTURE analysis. **a** ADMIXTURE results. **b** CV Errors for 5 replicates per K value. Source data are provided as a Source Data file.

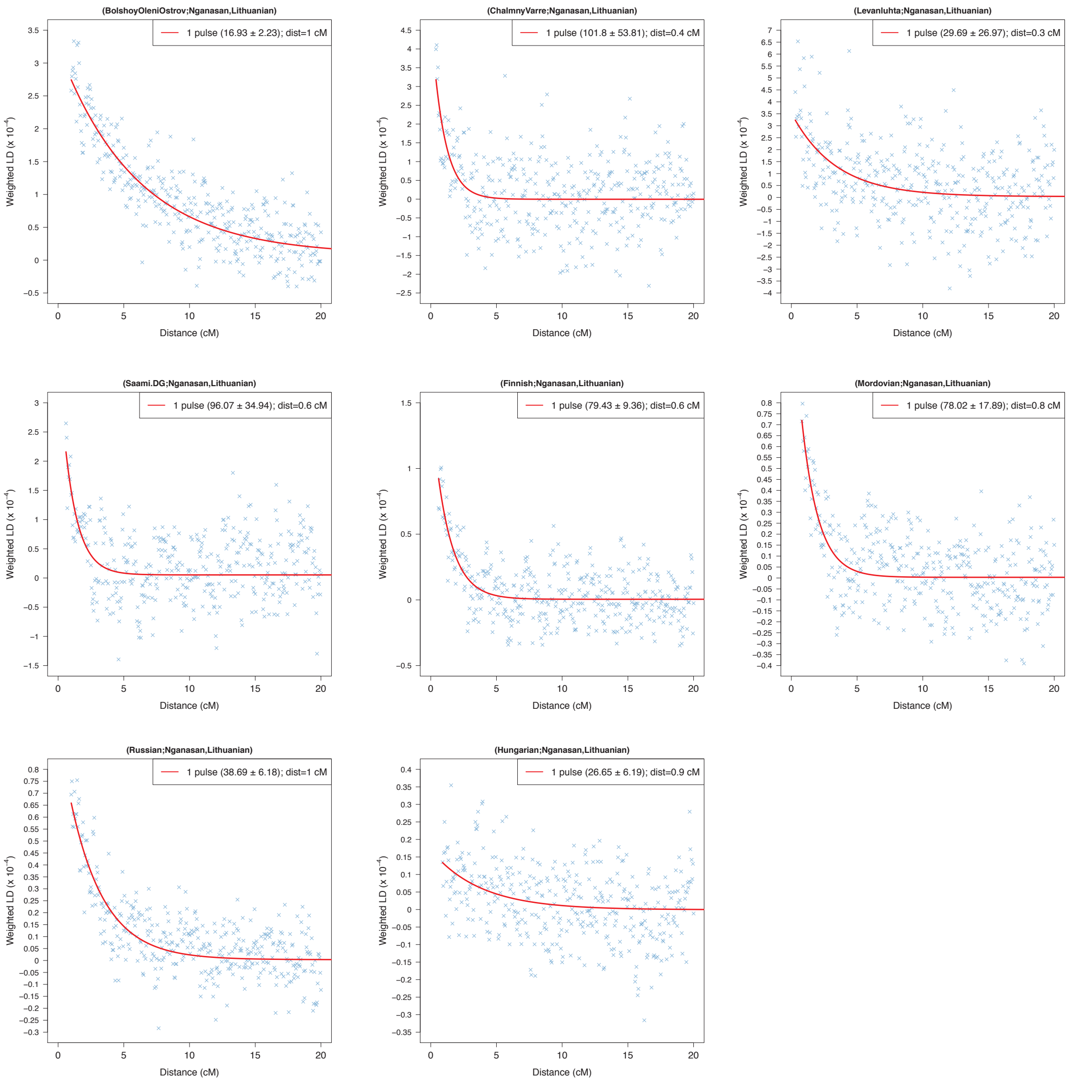

Supplementary Figure 5. Linkage Disequilibrium decay curves for ancient and modern populations. Minimum distance in cM used is the lowest distance for which ALDER provided results. Source data are provided as a Source Data file.

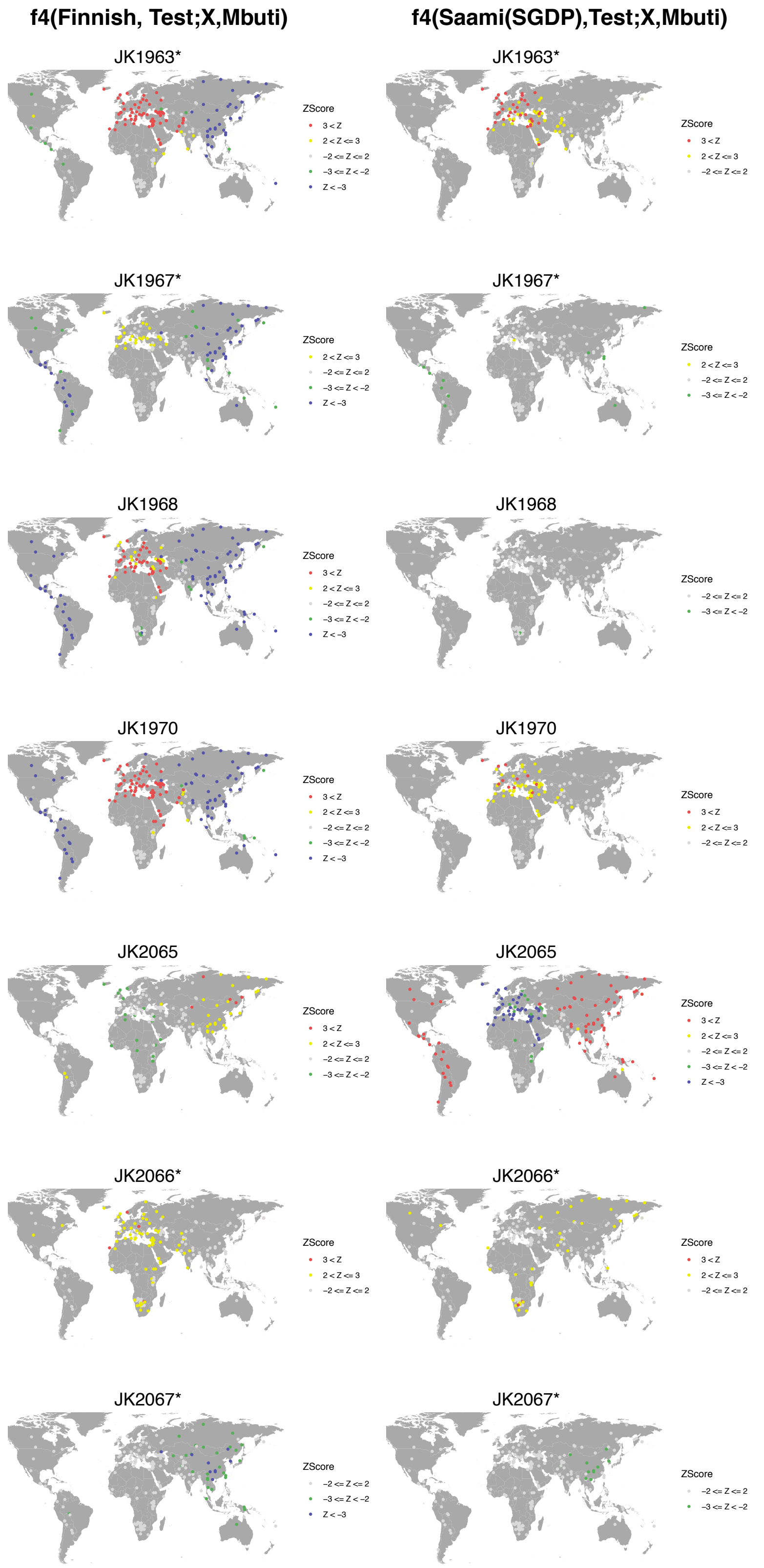

Supplementary Figure 6. Testing Levänluhta for cladality with modern Finns and/or Saami. f_4(Finnish, Test; X, Mbuti) & f_4(Saami, Test; X, Mbuti) comparison for multiple worldwide populations *X*, using ancient individuals from Levänluhta as *Test*. Asterisks denote individuals with low coverage (<15000 SNPs covered). Points are coloured based on bins of Z Score values, with warmer colours indicating higher affinity in Finns/Saami than in the *Test*, and vice versa for colder colours. Grey points indicate similar affinities toward population *X*. Map generated using ggplot2 and data from the Natural Earth project. Source data are provided as a Source Data file.

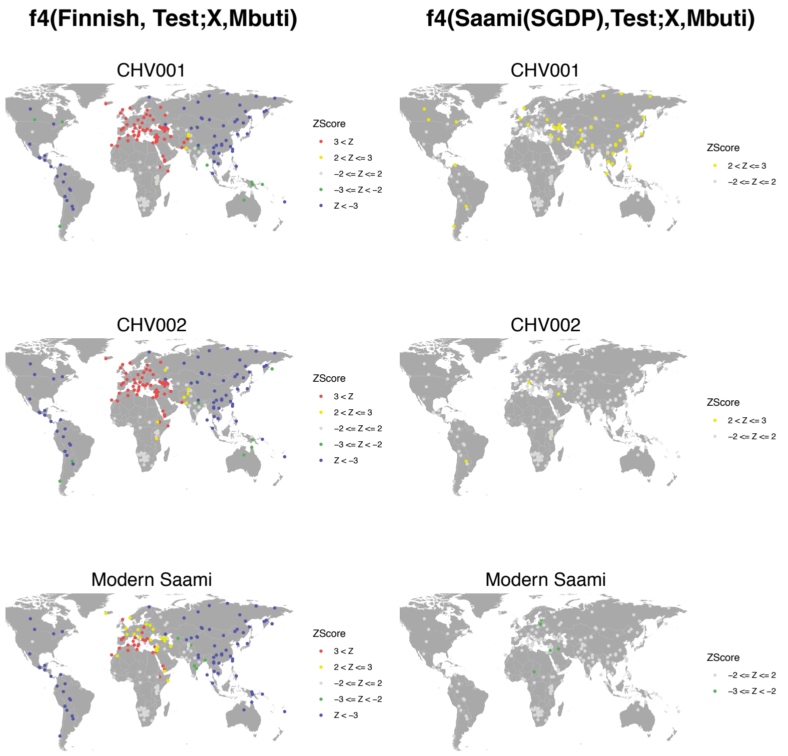

Supplementary Figure 7. Testing historical and modern Saami for cladality with modern Finns and/or Saami. f_4(Finnish, Test; X, Mbuti) & f_4(Saami, Test; X, Mbuti) comparison for multiple worldwide populations *X*, using ancient individuals from Chalmny Varre and the modern Saami genome from this study as *Test*. Points are coloured based on bins of Z Score values, with warmer colours indicating higher affinity in Finns/Saami than in the *Test*, and vice versa for colder colours. Grey points indicate similar affinities toward population *X*. Map generated using ggplot2 and data from the Natural Earth project. Source data are provided as a Source Data file.

Supplementary Tables

Supplementary Table 1. Deamination damage per library.

Library ID	UDG treatment	Damage 5’ 1st base	Damage 5’ 2nd base	Damage 3’ 2nd base	Damage 3’ 1st base
CHV001	Half	0.0287	0.0074	0.0121	0.031
CHV002	Half	0.0189	0.0062	0.0111	0.0244
JK1963	Half	0.0259	0.0071	0.0111	0.0294
JK1967	Half	0.0104	0.0048	0.009	0.0151
JK1968	Half	0.0284	0.0054	0.0088	0.0282
JK1968.nonUDG	None	0.094	0.0575	0.0521	0.0865
JK1970	Half	0.0239	0.0069	0.0109	0.029
JK1970.nonUDG	None	0.0953	0.0745	0.073	0.0937
JK2065	Half	0.0123	0.0045	0.0069	0.0155
JK2065.nonUDG	None	0.0471	0.0452	0.0448	0.0487
JK2066	Half	0.0146	0.0041	0.0059	0.0171
JK2067	Half	0.0104	0.004	0.0065	0.0129
BOO001	Half	0.0283	0.0075	0.0107	0.0302
BOO002	Half	0.0402	0.0078	0.0113	0.0333
BOO003	Half	0.0411	0.0073	0.0108	0.0398
BOO004	Half	0.0382	0.0085	0.0117	0.0344
BOO005	Half	0.045	0.0066	0.0108	0.0421
BOO006	Half	0.0381	0.0063	0.0094	0.0302

Supplementary Note 1

Extended Archaeological Information

Levänluhta

The Levänluhta site is located in the Isokyrö municipality at the southern Ostrobothnia region of Western Finland. The site represents a rarely observed case of lake burials, and is one of the most studied archaeological sites in Finland. Levänluhta context has been dated to the Iron Age in Finland (300-800 CE)[1,2] via some prestige artifacts assumed to have served as grave goods. The skeletal remains are, while numerous and well preserved, also anatomically disarticulated due to the gradual transition of the original lake environment to a marshland, and subsequent ditching and ploughing of the soil for agricultural use over the centuries. The remains of approximately 100 individuals are recognized from the cemetery to date.
The archaeological excavations were carried out by Oscar Rancken in 1886, A.M. Tallgren and Alfred Hackman from 1912 to 1913, Aarni Erä-Esko from the National Board of Antiquities from 1982 to 1984[1,2], followed by an archaeological survey of both Levänluhta and the immediate area around it in 2014. A comprehensive osteological analysis was reported by Tarja Formisto in 1993. The human remains are under the care of National Board of Antiquities, and stored currently at the National Museum of Finland. Skeletal element IDs for the analysed samples are: 2:1:a29 -L21 (JK1963), "milk tooth" -L47 (JK1967), 2:1:a16 -L20 (JK1968), 477 -L46 (JK1970), 2:1:a3 -L17 (JK2065), KM21814:735 -L22 (JK2066), 2:1:a2 -L16 (JK2067).

Chalmny Varre

The Chalmny-Varre Saami cemetery, associated with the two seasonal settlements of nomadic Kamensk Saami in the 18th century, is located on a small island in the middle flow of Ponoy River (center of Kola Peninsula). The burials have characteristics of Christian graves, combined with old traditional Saami rituals, such as birch cork pieces placed in the graves and masks (lichiny) of deceased carved on wooden crosses. Archaeological dating is confirmed by artifact findings from the graves and information on the wooden crosses which are marking the graves. The excavation, including an anthropological investigation, was organised by the Institute of Ethnography of N.N. Miklukho-Maclay Academy of Science of the USSR in the year 1976. Skeletal IDs for analysed samples are: CHV30 (CHV001), CHV38 (CHV002).

Bolshoy Oleniy Ostrov

Bolshoy Oleniy Ostrov (Great Reindeer Island), situated in the Kola Bay of the Barents Sea and separated from the mainland by Yekarerininsky Island and two straits, harbors the ancient cemetery of an unknown Early Metal Age culture. The preservation of artifacts made from bone and antler, wooden structures, as well as human remains is remarkable for the location and age this site represents. Altogether 19 skeletons of adults and children have been recognized from both single and collective burials of the site, together with more than 250 artifacts. Archaeological

surveys and excavations at the location were performed by G.D. Richter and S.F. Yegorov in 1925, by A.V. Schmidt on the USSR Academy of Science Kola Expedition in 1928, by N.N. Gurina in 1947–1948, as a part of Kola Expedition from the Leningrad Department of the Institute of Archaeology of the Academy of Sciences, and by V.Y. Shumkin from the same institute, later named as the Institute for the History of Material Culture Russian Academy of Sciences (RAS), starting from 1998-1999 and continuing in 2001-1004. Apart from these excavations, approximately 25 burials were revealed in 1934 during the construction of fortifications. Four finds are known to have been stored by the USSR Academy of Sciences at the time, but the location of all other remains from this instance is unknown. Part of the cemetery was never excavated and has possibly been destroyed by erosion. Morphological analyses, largely concentrated on cranial characteristics, have been performed by S.D. Sinitsyn in 1930, and V.P. Yakmov in 1953 and V.G. Moiseyev and V.I. Khartanovich in 2012[3]. A radiocarbon date for the site is provided by Moiseyev and Khartanovich as 3473±42 BP [3], which we calibrated as described in Methods. We note that radiocarbon dating on individuals with marine-based diets can be affected by the marine reservoir effect, which may result in overestimates of the true date[4]. Other dates provided by Murashkin et al (2016)[5] also date the site to the middle to late 2nd millennium BC. The human remains are stored at Peter the Great Museum of Anthropology and Ethnography (Kunstkamera) RAS in St. Petersburg, with the exception of burial 13, the remains of which are admitted to the Historical Museum in Polarnyi, Murmansk Oblast.[3] Skeleteal IDs for analysed samples are: BOO57.1 (BOO001), BOO72.1 (BOO002), BOO72.4 (BOO003), BOO72.7 (BOO004), BOO72.10 (BOO005), BOO72.15 (BOO006).

Supplementary Note 2

Assessment of power to detect contamination using continental supervised ADMIXTURE analysis.

To test the power of our approach to identify contamination using supervised ADMIXTURE using a set of continental populations as defined clusters, we devised a way to contaminate our data in silico. We assume that the rate of nuclear contamination in a library will be directly related to the proportion of reads mapping to the nuclear chromosomes that are of contaminant origin. Therefore, when a random draw genotyping approach is used, that contamination rate should also be directly proportional to the rate of genotypes called from the reads of the contaminant source. This assumption is violated if the distribution of contaminant and ancient DNA fragments across the nuclear genome is not uniform.

As a means to contaminate genotypes in silico, we provide ContaminateGenotypes.py (https://github.com/TCLamnidis/ContaminateGenotypes), a python script that contaminates the genotypes of a set of Sample individuals with genotypes from a provided contaminant individual, at multiple specified rates. We used this script to create a set of dummy individuals contaminated by one of seven contaminant individuals at nine different contamination rates.

As contamination sources we provided one individual from each population used as a pre-defined continental cluster during the supervised ADMIXTURE analysis (Atayal: NA13597, French: HGDP00511, Kalash: HGDP00267, Karitiana: HGDP00995, Mbuti: HGDP00449, Papuan: HGDP00540). We additionally used one Han Chinese individual (HGDP00774) as a contaminant to assess the power of this analysis to identify contamination that is not within the pre-defined continental clusters.

We generated dummy individuals at contamination rates of 0.01, 0.02, 0.03, 0.04, 0.05, 0.08, 0.10, 0.25, 0.50 for each of the contaminant sources. The dummy individuals were then separated by contaminant source and contamination rate, before running supervised admixture on each subset separately, to avoid artefacts in ADMIXTURE from essentially clonal individuals.

Our results show that contaminants that are distantly related to the ancestries within the ancient individuals (e.g. Mbuti and Papuan in our case), are detected from contamination rates of 0.05 and above, while more closely related ancestries, though ones not present in the dataset (e.g. Kalash) can be detected less consistently at that threshold, but quite consistently at contamination rates of 0.08 and above (Supplementary Figure 8).

Finally, contamination from sources whose continental ancestry was already detected in the uncontaminated data (e.g. Atayal, French, Karitiana) cannot be reliably detected as such (Supplementary Figure 9). We conclude that such an approach only has power to detect contamination from distantly related populations.

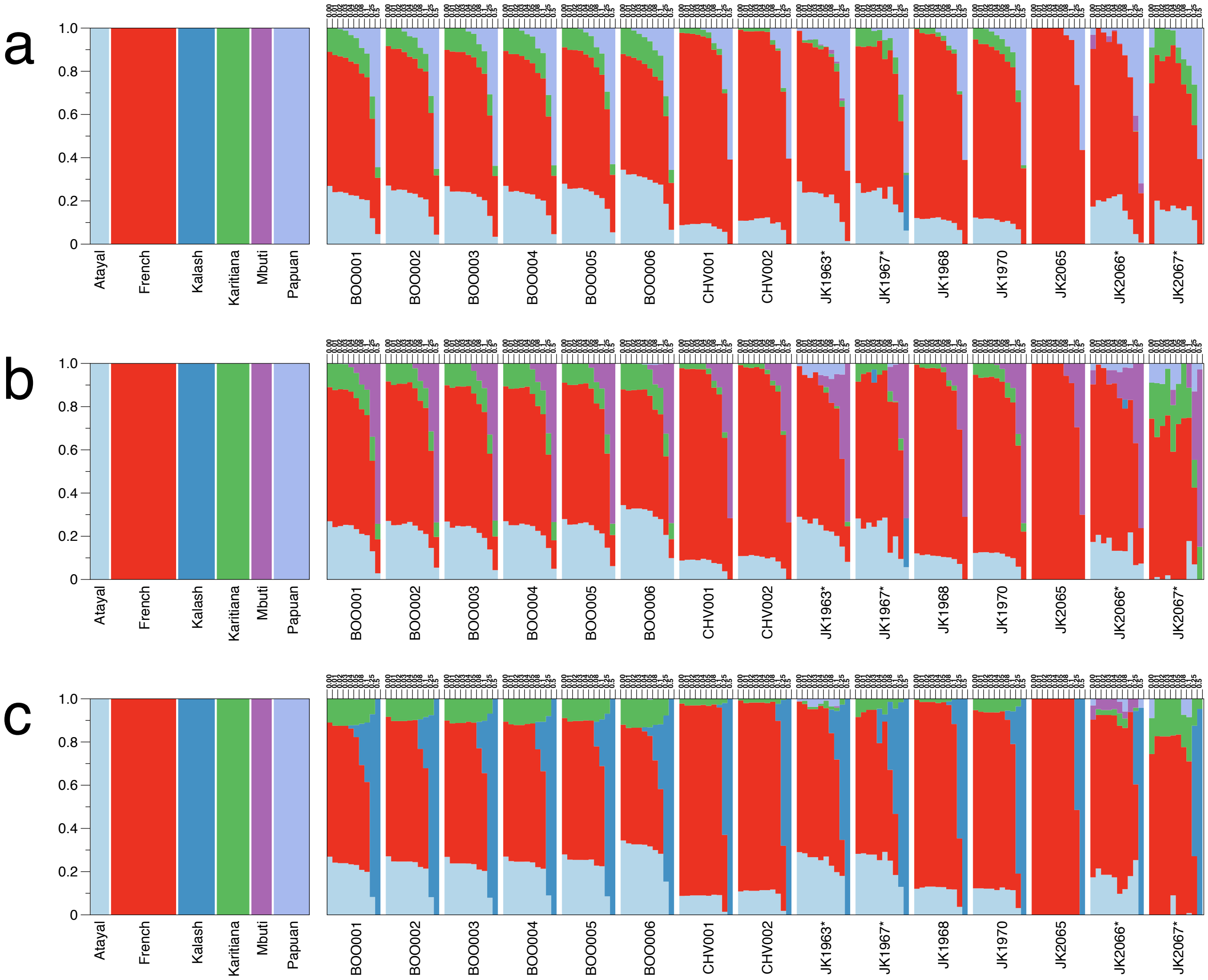

Supplementary Figure 8. Compiled supervised ADMIXTURE runs. Each individual from this study was contaminated at different rate by a distantly related contaminant individual. Low coverage (>15,000 SNPs) individuals marked with an asterisk. **a** Contaminant was Papuan. **b** Contaminant was Mbuti. **c** Contaminant was Kalash. Source data are provided as a Source Data file.

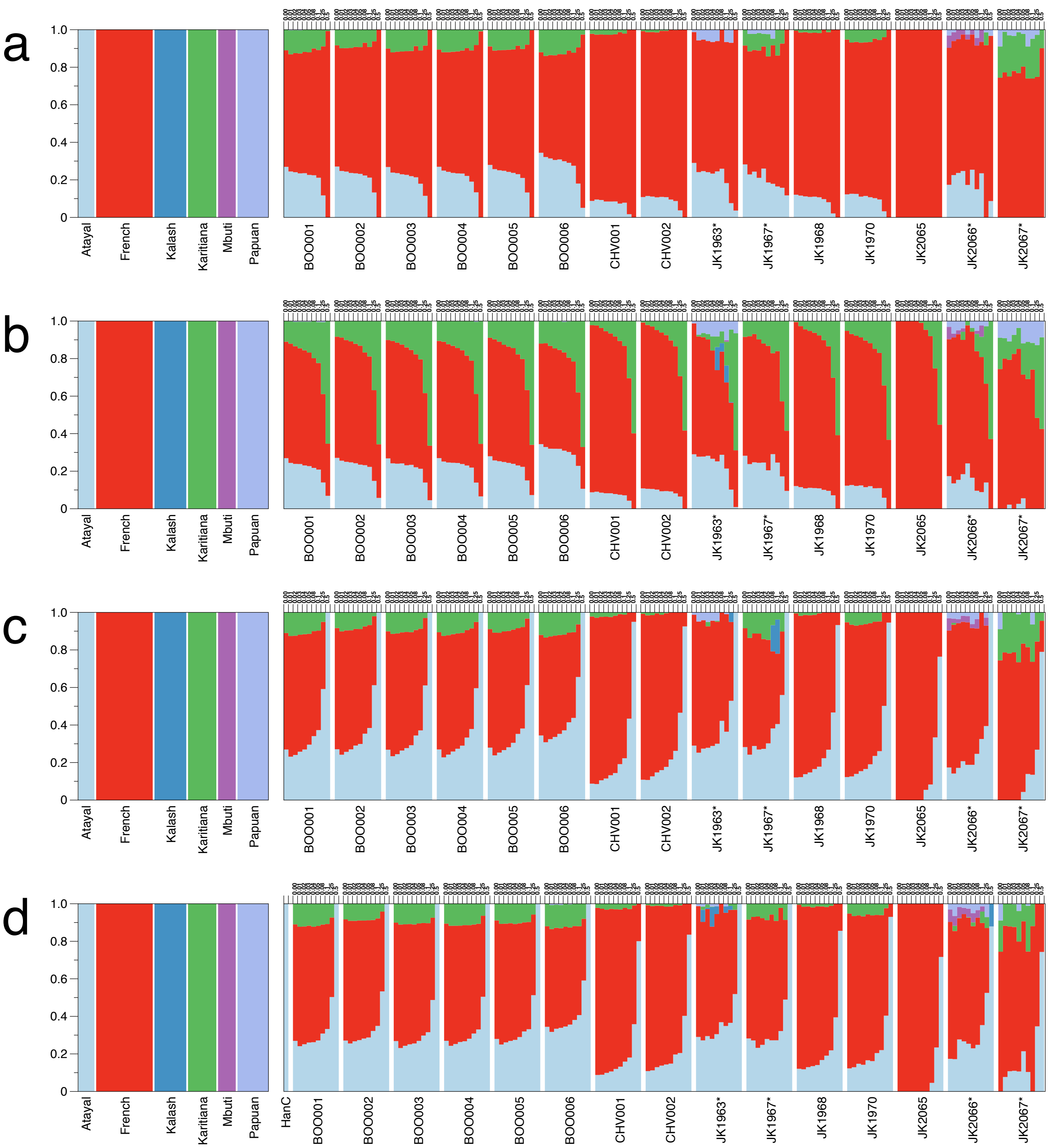

Supplementary Figure 9. Compiled supervised ADMIXTURE runs. Each individual from this study was contaminated at different rate by a contaminant individual from a genetic cluster that was already present in the uncontaminated data. Low coverage (>15,000 SNPs) individuals marked with an asterisk. **a** Contaminant was French. **b** Contaminant was Karitiana. **c** Contaminant was Atayal. **d** Contaminant was Han Chinese. Contaminant individual is labelled "HanC". Source data are provided as a Source Data file.

Supplementary Note 3

Sex determination with error bar calculation

We provide a python script (https://github.com/TCLamnidis/Sex.DetERRmine.git) that calculates the relative coverage of X and Y chromosomes, and their associated error bars, from the depth of coverage at specified SNPs. The error calculation relies on the assumption that reads are randomly and independently distributed across a list of specified SNPs. This is justified if SNPs are further apart from each other than typical sequencing length, which is largely the case for the SNP panel considered here (1240K). The script takes as input the output of the samtools depth command, which reports the depth at each of the specified SNPs. The depths are partitioned into three bins: Autosomal, X- and Y-chromosome, so that:

$$N = \sum_i N_i$$

Where N_i is the number of sequenced reads overlapping the SNPs within each bin i, and N is the total number of reads overlapping with SNPs within our capture panel.
We can then calculate the proportion of all sequenced reads that cover SNPs in bin i (p_i). Its associated error is then calculated as the error of the binomial distribution of N_i.

$$p_i = \frac{N_i}{N}$$

$$\mathrm{Err}(N_i) = \sqrt{N p_i (1 - p_i)}$$

The average SNP depth within bin i (d_i) can be calculated as the ratio of N_i to the number of SNPs within bin i (S_i). The error of the average SNP depth is proportional to the error associated with N_i.

$$d_i = \frac{N_i}{S_i}$$

$$\mathrm{Err}(d_i) = \frac{\mathrm{Err}(N_i)}{S_i}$$

The relative coverage on the X and Y chromosomes can then be calculated as the ratio of the average SNP depth on the X/Y chromosome over the average SNP depth on the autosomes. The error of this measurement can then be calculated using standard error propagation.

$$\mathrm{rate}_{X/Y} = \frac{d_{X/Y}}{d_{aut}}$$

$$\mathrm{Err}(\mathrm{rate}_{X/Y}) = \sqrt{\left(\mathrm{Err}(d_{X/Y}) \times \frac{1}{d_{aut}}\right)^2 + \left(\mathrm{Err}(d_{aut}) \times \frac{d_{X/Y}}{{d_{aut}}^2}\right)^2}$$

Supplementary References

1. Wessman, A. Levänluhta. A place of punishment, sacrifice or just a common cemetery? *Fennoscandia archaeologica* **XXVI,** 81–105 (2009).
2. Wessman, A. *et al.* Hidden and Remote: New Perspectives on the People in the Levänluhta Water Burial, Western Finland (c.ad 300–800). *European Journal of Archaeology* **21,** 431–454 (2018).
3. Moiseyev, V. G. & Khartanovich, V. I. Early Metal Age crania from Bolshoy Oleniy Island, Barents Sea. *Archaeology, Ethnology and Anthropology of Eurasia* **40,** 145–154 (2012).
4. Ascough, P., Cook, G. & Dugmore, A. Methodological approaches to determining the marine radiocarbon reservoir effect. *Progress in Physical Geography: Earth and Environment* **29,** 532–547 (2005).
5. Murashkin, A. I., Kolpakov, E. M., Shumkin, V. Y., Khartanovich, V. I. & Moiseyev, V. G. Kola Oleneostrovskiy grave field: a unique burial site in the European Arctic. *New Sites, New Methods; Iskos 21* (2016).
