# Supplementary material for: Ancient Fennoscandian genomes reveal origin and spread of Siberian ancestry in Europe
Source: Nat Commun. 2018 Nov 27;9:5018. doi: 10.1038/s41467-018-07483-5 (PMC6258758; doi:10.1038/s41467-018-07483-5)
Supplement: Supplementary file 3 — Description of Additional Supplementary Files [file 41467_2018_7483_MOESM3_ESM.pdf]

The legends of the Supplementary Data files are:
Supplementary Data 1. Information on comparative dataset used for this study.

Supplementary Data 2. Phenotypic SNP analysis. The associated gene, chromosome, position, rs tag and observed alleles are shown. For each studied individual, the number of reads supporting each allele is shown as a pair x/y where x denotes the number of reads supporting allele 1, and y the number of reads supporting allele 2.

Supplementary Data 3. f3(Test; Siberian source, European source). the f3 with the most negative Z Score is highlighted in bold for each populations, where applicable.

Supplementary Data 4. qpAdm models. Models with p-values p≥0.05, 0.05>p≥0.01 and 0.01>p are highlighted in green, yellow and red respectively. Individuals from this study and populations containing them are shown in bold.

Supplementary Data 5. f4 (Finnish, Test; X, Mbuti) for multiple worldwide populations X. Z Scores highlighted by significance bin. Red: Z>3, yellow: 3≥Z>2, gray: 2≥Z≥-2, green: -2>Z≥-3 and blue: Z<-3.

Supplementary Data 6. f4 (Saami (SGDP), Test; X, Mbuti) for multiple worldwide populations X. Z Scores highlighted by significance bin. Red: Z>3, yellow: 3≥Z>2, gray: 2≥Z≥-2, green: -2>Z≥-3 and blue: Z<-3.

Yours sincerely, on behalf of all authors,
Stephan Schiffels
